# Geometric Characteristics of the Wasserstein Metric on SPD(n) and Its Applications on Data Processing

**DOI:** 10.3390/e23091214

**Published:** 2021-09-14

**Authors:** Yihao Luo, Shiqiang Zhang, Yueqi Cao, Huafei Sun

**Affiliations:** 1 School of Mathematics and Statistics, Beijing Institute of Technology, Beijing 100081, China; knowthingless@bit.edu.cn; 2 Department of Computing, Imperial College London, London SW7 2AZ, UK; s.zhang21@imperial.ac.uk; 3 Department of Mathematics, Imperial College London, London SW7 2AZ, UK; y.cao21@imperial.ac.uk

**Keywords:** symmetric positive-definite matrix, Wasserstein metric, curvature, point cloud denoising, image edge detecting

## Abstract

The Wasserstein distance, especially among symmetric positive-definite matrices, has broad and deep influences on the development of artificial intelligence (AI) and other branches of computer science. In this paper, by involving the Wasserstein metric on SPD(n), we obtain computationally feasible expressions for some geometric quantities, including geodesics, exponential maps, the Riemannian connection, Jacobi fields and curvatures, particularly the scalar curvature. Furthermore, we discuss the behavior of geodesics and prove that the manifold is globally geodesic convex. Finally, we design algorithms for point cloud denoising and edge detecting of a polluted image based on the Wasserstein curvature on SPD(n). The experimental results show the efficiency and robustness of our curvature-based methods.

## 1. Introduction

Symmetric positive-definite matrices have wide usage in many fields of information science, such as stability analysis of signal processing, linear stationary systems, optimal control strategies and imaging analysis [1,2,3]. Its importance is beyond words [4,5]. Instead of considering a single matrix, contemporary scientists tend to comprehend the global structure of the set consisting of all n×n symmetric positive-definite matrices. This set is known as SPD(n). SPD(n) can be endowed with various structures. The most traditional Euclidean metric is induced as submanifold metric from the Euclidean inner product on the space of matrices. X. Pennec, P. Fillard et al. [6] defined the affine-invariant Riemannian metric. V. Arsigny, P. Fillard et al. [7] showed the Lie group [8] structure on SPD(n), which admits a bi-invariant metric called the Log-Euclidean metric.

Recently, by constructing a principle bundle, Y. Li, M. Wong et al. [9] and S. Zhang et al. [10] defined a new Riemannian metric on SPD(n) whose geodesic distance is equivalent to the Wasserstein-2 [11,12] distance, the so-called Wasserstein metric. This distance rather than the metric has been widely used in artificial intelligence [13]. In geometry, encouragingly, T. Asuka [14] and E. Massart, P.-A. Absil [15] gave a series of expressions for geometric quantities theoretically. However, these expressions are too general or complicated to be applied. In this paper, we derive more computationally feasible expressions in a concrete case. Moreover, we give the Jacobi field and scalar curvature.

Along the blooming of data science, point cloud processing, especially denoising, plays an increasingly important role in data relevant researches and engineering. There are immense literature in point cloud denoising and widely used algorithms packed as inline functions of softwares such as PCL [16]. These methods share a common drawback when high density noise is added to point clouds. Utilizing the geometric structure of SPD(n), we design a novel algorithm to overcome this drawback. Compared to traditional methods, our algorithm is more accurate and less dependent on artificial parameters.

In addition to that, we involve our theory for image edge detection, which is a classical problem in image processing and design a new detecting algorithm. Different from traditional gradient-based filters, such as Sobel, Prewitt and Laplacian, we present the connection between Wasserstein sectional curvature and edges. Experiments show the feasibility of our algorithm.

The paper is organized as follows. In Section 2, we introduce some basic knowledge of the Riemannian manifold $\left(SPD\left(n\right),g_{W}\right)$, and consider the symmetry of the $\left(SPD\left(n\right),g_{W}\right)$ as well. In Section 3, we describe the Wasserstein geometry of SPD(n), including the geodesic, exponential map, connection and curvature. In particular, we prove the geodesic convexity and the nonexistence of cut locus and conjugate pair. In Section 4, we design an adaptive algorithm to denoise point clouds. In Section 5, we develop a curvature-based method to detect image edge. Proofs and detailed numerical results are presented in the Appendix B.

## 2. Preliminary

### 2.1. Notation

In this paper, we adopt conventional notations in algebra and geometry. Riemannian manifolds are denoted as pairs of (manifold,metric). For example, our mainly interesting object is $\left(SPD\left(n\right),g_{W}\right)$, meaning SPD(n) endowed with Wasserstein metric. $R^{n}$ is the *n*-dimensional Euclidean space. M(n) represents the set of n×n matrices, Sym(n) means the set of n×n symmetric matrices, and O(n) means the set of n×n orthogonal matrices. $T_{A}M$ is conventionally the tangent space of *M* at a point *A*.

Λ always represents a diagonal n×n matrix. For an n×n matrix *A*, λ(A) or $\lambda_{i}\left(A\right)$ means an eigenvalue or the *i*-th eigenvalue of *A*, respectively. The components of matrix *A* with the entries $A_{ij}$ will always be noted as $\left[A_{ij}\right]$. The identity matrix is denoted as *I*. In this paper, we conventionally express points on manifolds as A,B and vector fields as X,Y.

Sylvester equation is one of the most classical matrix equations. The following special case of Sylvester equation plays a key role in understand the geometry of $\left(SPD\left(n\right),g_{W}\right)$

(1) $$\begin{array}{c} AK+KA=X,A\in SPD(n),X\in Sym(n). \end{array}$$

We denote the solution about *K* of (1) as $\Gamma_{A}\left[X\right]$. Then, the matrix $\Gamma_{A}\left[X\right]\in M\left(n\right)$ satisfies

(2) $$\begin{array}{c} A\Gamma_{A}\left[X\right]+\Gamma_{A}\left[X\right]A=X. \end{array}$$

From geometric aspects, we can ensure the existence and uniqueness of the solution in the case involved in this paper. Some properties of $\Gamma_{A}\left[X\right]$ can be found in Appendix A. More details about the Sylvester equation are presented in [17].

We recall an algorithm to solve this kind of Sylvester equations, which offers an explicit expression of the solution. This expression only depends on the eigenvalue decomposition. More details can be found in [10].

Algorithm 1 will be used frequently in the following passage, and it helps us to comprehend the geometry of SPD(n). Note that this algorithm can also be used for general X∈M(n). In particular, when *X* is symmetric (skew-symmetric), $\Gamma_{A}\left[X\right]$ is also symmetric (skew-symmetric). Moreover, this algorithm will be simplified if *A* is diagonal.
**Algorithm 1** Solution of Sylvester Equation.**Input:**A∈SPD(n),X∈Sym(n)**Output:**$\Gamma_{A}\left[X\right]$1:Eigenvalue decomposition, $A=Q\Lambda Q^{T}$, where $Q\in O\left(n\right),\Lambda :=\mathrm{diag}\left(\lambda_{1},\cdots ,\lambda_{n}\right)$ are eigenvalues of *A*;2:$C_{X}:=\left[c_{ij}\right]=Q^{T}XQ$;3:$E_{X}=\left[e_{ij}\right]=\left(\frac{c_{ij}}{\lambda_{i}+\lambda_{j}}\right)$;4:$\Gamma_{A}\left[X\right]=QEQ^{T}$.

### 2.2. Wasserstein Metric

In this part, we introduce the Wasserstein metric on SPD(n).

**Definition** **1.** *For any A∈SPD(n), $X,Y\in T_{A}SPD\left(n\right)$, define*

(3) $$\begin{array}{c} g_{W}{|}_{A}\left(X,Y\right)=\mathrm{tr}\left(\Gamma_{A}\left[X\right]A\Gamma_{A}\left[Y\right]\right)=\frac{1}{2}\mathrm{tr}\left(X\Gamma_{A}\left[Y\right]\right). \end{array}$$

In the second equation, we use the facts that $\Gamma_{A}\left[X\right],\Gamma_{A}\left[Y\right],A$ are all symetric and that $A\Gamma_{A}\left[X\right]+\Gamma_{A}\left[X\right]A=X$. One can check that $g_{W}$ is a symmetric and non-degenerated bilinear tensor fields on SPD(n) [18]. We call $g_{W}$ the Wasserstein metric.

Denote $g_{E}\left(\tilde{X},\tilde{Y}\right):=\mathrm{tr}\left({\tilde{X}}^{T}\tilde{Y}\right),\forall \tilde{A}\in GL\left(n\right),\tilde{X},\tilde{Y}\in T_{\tilde{A}}GL\left(n\right)$ as Euclidean metric on GL(n). Then, we have the following conclusions.

**Proposition** **1.** *The projection*

(4) $$\begin{array}{cc} \sigma :(GL\left(n\right),g_{E}) & \to (SPD\left(n\right),g_{W})\\ \tilde{A} & \mapsto {\tilde{A}}^{T}\tilde{A} \end{array}$$

*is a Riemannian submersion [19], which means that dσ is surjective and*

(5) $$\begin{array}{c} g_{E}\left(\tilde{X},\tilde{Y}\right)=g_{W}\left(d\sigma \left(\tilde{X}\right),d\sigma \left(\tilde{Y}\right)\right)=g_{W}\left(X,Y\right). \end{array}$$

**Remark** **1.** *The general linear group with Euclidean metric $\left(GL\left(n\right),g_{E}\right)$ and projection σ is a trivial principal bundles on SPD(n) with orthogonal group O(n) as the structure group. This bundle structure establishes two facts [10]: SPD(n)≅GL(n)/O(n), and $g_{E}$ remains invariant under the group action of O(n).*

Before giving more conclusions, we review some concepts. For any $\tilde{A}\in GL\left(n\right)$, we say that the tangent vector $\tilde{V}\in T_{\tilde{A}}GL\left(n\right)$ is vertical if $d\sigma (\tilde{V})=0$, and $\tilde{W}\in T_{\tilde{A}}GL\left(n\right)$ is horizontal if $g_{W}{|}_{\tilde{A}}\left(\tilde{V},\tilde{W}\right)=0$ for all vertical vecters $\tilde{V}$. In addition to that, if $d\sigma \left(\tilde{X}\right)=X\in T_{A}SPD\left(n\right)$, we call $\tilde{X}$ as a lift of *X*, where $A=\sigma (\tilde{A})$. Using the notation $\Gamma_{A}\left[X\right]$, we can find the horizontal lift of $X\in T_{A}SPD\left(n\right)$.

**Proposition** **2.** *For any $\tilde{A}\in \left(GL\left(n\right),g_{E}\right)$, $A=\sigma (\tilde{A})$ and any $X\in T_{A}SPD\left(n\right)$, there is a unique $\tilde{X}$ to be the horizontal lift of X at $T_{\tilde{A}}GL\left(n\right)$—that is,*

(6) $$\begin{array}{c} \tilde{X}=\tilde{A}\Gamma_{A}\left[X\right]. \end{array}$$

Using Proposition 2, we can obtain the representations of horizontal and vertical vectors.

**Proposition** **3.** *For any $\tilde{A}\in \left(GL\left(n\right),g_{E}\right)$, $T_{\tilde{A}}GL\left(n\right)$ has the following orthogonal decomposition*

(7) $$\begin{array}{c} T_{\tilde{A}}GL\left(n\right)=H\left(\tilde{A}\right)⊕V\left(\tilde{A}\right), \end{array}$$

*where $H(\tilde{A})$ consists of all horizontal vectors, $V(\tilde{A})$ consists of all vertical vectors, and*

(8) $$\begin{array}{c} H\left(\tilde{A}\right)=\left\{\tilde{A}K\;|\;K^{T}=K\right\},V\left(\tilde{A}\right)=\left\{S\tilde{A}\;|\;S^{T}=-S\right\}. \end{array}$$

Proofs of Proposition 2 and 3 can be found in [10].

### 2.3. Symmetry

Now we study the symmetry of $\left(SPD\left(n\right),g_{W}\right)$. Consider orthogonal group action Ψ:O(n)×SPD(n)→SPD(n) defined by

(9) $$\begin{array}{c} \Psi_{O}\left(A\right)=OAO^{T},\forall O\in O\left(n\right),A\in SPD\left(n\right). \end{array}$$

One can check that Ψ is a group action of O(n) and that $d\Psi_{O}$ are isometric for all O∈O(n), which means that O(n) is isomorphic to a subgroup of the isometry group $ISO(SPD\left(n\right),g_{W})$ on SPD(n). Precisely, we have

(10) $$\begin{array}{c} {\left\{\Psi_{O}\right\}}_{O\in O(n)}⊲ISO\left(SPD\left(n\right),g_{W}\right). \end{array}$$

According to (10), when we study local geometric characteristics, we only need to consider the sorted diagonal matrices as the representational elements under the orthogonal action rather than all general points on SPD(n). Therefore, some pointwise quantities, such as the scalar curvature and the bounds of sectional curvatures, depend only on the eigenvalues.

At the end of this part, we give the symmetry degree of $\left(SPD\left(n\right),g_{W}\right)$, which is defined by the dimension of $ISO(SPD\left(n\right),g_{W})$.

**Proposition** **4.** *$\left(SPD\left(n\right)\right),g_{W})$ has its symmetry degree controlled by*

(11) $$\begin{array}{c} \frac{1}{2}\left(n-1\right)n\leq \dim\left(ISO\left(SPD\left(n\right),g_{W}\right)\right)\leq \frac{1}{8}\left(n-1\right)n\left(n+1\right)\left(n+2\right)+1. \end{array}$$

**Proof.**  The famous interval theorem [20] about isometric group shows the nonexistence of isometric groups with dimension between $\frac{m(m-1)}{2}+1$ and $\frac{m(m+1)}{2}$, for any *m*-dimensional Riemannian manifold, except m≠4.

On one hand, For an *n*-dimensional Riemannian manifold, the dimension of isometry group achieves maximum if and only if it has constant sectional curvature. However, we will show later that $\left(SPD\left(n\right),g_{W}\right)$ has no constant sectional curvature, which means its symmetry degree is less than the highest.

On the other hand, Equation (10) shows that the dimension of Wasserstein isometric group is higher than the dimension of O(n). Therefore, by $\dim\left(SPD\left(n\right)\right)=\frac{n^{2}+n}{2}\neq 4$ and $\dim\left(O\left(n\right)\right)=\frac{n^{2}-n}{2}$, we obtain the desired result.    □

## 3. Wasserstein Geometry of SPD(n)

### 3.1. Geodesic

In this part, we give the expression of geodesic on $\left(SPD\left(n\right),g_{W}\right)$, including the geodesic jointing of two points and the geodesic with initial values. Then, we will show that the whole Riemannian manifold $\left(SPD\left(n\right),g_{W}\right)$ is geodesic convex, which means that we can always find the minimal geodesic jointing any two points. To some extent, geodesic convexity may make up for the incompleteness of $\left(SPD\left(n\right),g_{W}\right)$.

To prove the geodesic convexity of $\left(SPD\left(n\right),g_{W}\right)$, we need the following theorem.

**Theorem** **1.** *For any $A_{1},A_{2}\in SPD\left(n\right)$, let ${\tilde{A}}_{1}=A_{1}^{\frac{1}{2}}$ be the fixed lift of $A_{1}$, there exists a lift of $A_{2}$*

(12) $$\begin{array}{c} {\tilde{A}}_{2}=A_{1}^{-\frac{1}{2}}{\left(A_{1}A_{2}\right)}^{\frac{1}{2}}, \end{array}$$

*such that the line segment $\tilde{\gamma }\left(t\right)=t{\tilde{A}}_{2}+\left(1-t\right){\tilde{A}}_{1},t\in \left[0,1\right]$ is horizontal and non-degenerated.*

Proof of Theorem 1 can be found in Appendix B. This theorem brings some geometrical and physical facts.

**Corollary** **1.** *(geodesic convexity) $\left(SPD\left(n\right),g_{W}\right)$ is a geodesic convex Riemannian manifold. Between any two points $A_{1},A_{2}\in SPD\left(n\right)$, there exists a minimal Wasserstein geodesic*

(13) $$\begin{array}{c} \gamma \left(t\right)={\left(1-t\right)}^{2}A_{1}+t\left(1-t\right)\left({\left(A_{1}A_{2}\right)}^{\frac{1}{2}}+{\left(A_{2}A_{1}\right)}^{\frac{1}{2}}\right)+t^{2}A_{2}, \end{array}$$

*where γ(t) lies on SPD(n) strictly. Thus, $\left(SPD\left(n\right),g_{W}\right)$ is geodesic convex.*

The similar expression of geodesic can also be found in several papers [14,15].

**Theorem** **2.** *For any two points in $\left(SPD\left(n\right),g_{W}\right)$, there exists a unique geodesic jointing them. From geometric aspect, there is no cut locus on any geodesic.*

**Proof.**  We have proved the existence of minimal geodesic jointing any two points in Corollary 1. Assume that the there exists two minimal geodesics jointing A,B∈SPD(n). Fix $\tilde{A}=A^{\frac{1}{2}}$ as the horizontal lift of *A* and lift horizontally these two geodesic, we will find two horizontal lifts of *B*. Denote these two lifts as ${\tilde{B}}_{1}=B^{\frac{1}{2}}Q_{1},{\tilde{B}}_{2}=B^{\frac{1}{2}}Q_{2},Q_{1},Q_{2}\in O\left(n\right)$. Then, $Q_{1}$ and $Q_{2}$ are both solutions of the following optimization problem

(14) $$\begin{array}{c} \underset{Q\in O(n)}{\arg\min}d_{F}\left(A^{\frac{1}{2}},B^{\frac{1}{2}}Q\right). \end{array}$$

Since the compactness of O(n), this problem has a unique solution. Thus, we prove the uniqueness of minimal geodesic.    □

**Remark** **2.** *Due to the nonexistence of cut locus, there exists no conjugate pair on $\left(SPD\left(n\right),g_{W}\right)$.*

### 3.2. Exponential Map

Following Lemma A1, we can directly write down the Wasserstein logarithm on SPD(n), for any $A_{1}\in SPD\left(n\right)$, $\log_{A_{1}}:SPD\left(n\right)\to T_{A_{1}}SPD\left(n\right)$

(15) $$\begin{array}{c} \log_{A_{1}}A_{2}{=d\sigma |}_{A_{1}^{\frac{1}{2}}}\dot{\tilde{\gamma }}\left(0\right)={\left(A_{1}A_{2}\right)}^{\frac{1}{2}}+{\left(A_{2}A_{1}\right)}^{\frac{1}{2}}-2A_{1}. \end{array}$$

By solving the inverse problem of above equation, we gain the expression of the Wasserstein exponential.

**Theorem** **3.** *In a small open ball B(0,ε),ε>0 in $T_{A}SPD\left(n\right)≅R^{\frac{1}{2}n\left(n+1\right)}$, the Wasserstein exponential at A, $\exp_{A}:B\left(0,\varepsilon \right)\to SPD\left(n\right)$ is explicitly expressed by*

(16) $$\begin{array}{c} \exp_{A}X=A+X+\Gamma_{A}\left[X\right]A\Gamma_{A}\left[X\right]. \end{array}$$

**Proof.**  By choosing the normal coordinates [21] at *A*, there always exist neighborhoods where $\exp_{A}$ is well-defined. From (15), given $\exp_{A}X$ as well-defined, this satisfies

(17) $$\begin{array}{c} {\left(A\exp_{A}X\right)}^{\frac{1}{2}}+{\left(\exp_{A}XA\right)}^{\frac{1}{2}}=X+2A. \end{array}$$

This equation can convert to the Sylvester equation, and we can express its solution as

(18) $$\begin{array}{cc} \; & AA^{-1}{\left(A\exp_{A}X\right)}^{\frac{1}{2}}+{\left(\exp_{A}XA\right)}^{\frac{1}{2}}A^{-1}A=X+2A\\ \Leftrightarrow \; & A\left(A^{-1}{\left(A\exp_{A}X\right)}^{\frac{1}{2}}\right)+\left(A^{-1}{\left(A\exp_{A}X\right)}^{\frac{1}{2}}\right)A=X+2A\\ \Leftrightarrow \; & A\Gamma_{A}\left[X+2A\right]={\left(A\exp_{A}X\right)}^{\frac{1}{2}}. \end{array}$$

Therefore, we have

(19) $$\begin{array}{c} \exp_{A}X=\Gamma_{A}\left[X+2A\right]A\Gamma_{A}\left[X+2A\right]=A+X+\Gamma_{A}\left[X\right]A\Gamma_{A}\left[X\right], \end{array}$$

which finishes this proof.    □

**Remark** **3.** *We call the first two terms of right hand A+X as the Euclidean exponential, and the last term of right hand $\Gamma_{A}\left[X\right]A\Gamma_{A}\left[X\right]$ as the Wasserstein correction for this bend manifold.*

**Corollary** **2.** *The geodesic equations with initial conditions $\gamma \left(0\right),\dot{\gamma }\left(0\right)$ on $\left(SPD\left(n\right),g_{W}\right)$ have the following explicit solution*

(20) $$\begin{array}{c} \gamma \left(t\right)=\gamma \left(0\right)+t\dot{\gamma }\left(0\right)+t^{2}\Gamma_{\gamma (0)}\left[\dot{\gamma }\left(0\right)\right]\gamma \left(0\right)\Gamma_{\gamma (0)}\left[\dot{\gamma }\left(0\right)\right],t\in \left(-\varepsilon ,\varepsilon \right). \end{array}$$

Using an exponential map, one can directly construct Jacobi fields with the geodesic variation.

**Theorem** **4.** *Along a geodesic γ(t) with $\gamma \left(0\right)=A\in SPD\left(n\right),\dot{\gamma }\left(0\right)=X\in T_{A}SPD\left(n\right)$, there exists a unique normal Jacobi vector field J(t) with initial conditions $J\left(0\right)=0,\nabla_{\dot{\gamma }\left(0\right)}J\left(t\right)=Y\in T_{A}SPD\left(n\right)$, where $g_{W}{|}_{A}\langle X,Y\rangle =0$. We have*

(21) $$\begin{array}{c} J\left(t\right)=tY+t^{2}\left(\Gamma_{A}\left[X\right]A\Gamma_{A}\left[Y\right]+\Gamma_{A}\left[Y\right]A\Gamma_{A}\left[X\right]\right). \end{array}$$

As in [18], J(t) is constructed by

(22) $$\begin{array}{c} J\left(t\right):={\left.\frac{\partial }{\partial s}\right|}_{s=0}\exp_{A}t\left(X+sY\right), \end{array}$$

substituting (16) into (22), and Theorem 4 comes from direct computation.

Subsequently, the next natural question is what is the maximal length of the extension of a geodesic. This question is equivalent to what is the largest domain of the exponential. We still focus on diagonal matrices.

**Theorem** **5.** *For any A∈SPD(n) and $X\in T_{A}SPD\left(n\right)$, $\exp_{A}\left(tX\right):\left[0,\varepsilon \right)\to SPD\left(n\right)$ is well-defined if and only if*

(23) $$\begin{array}{c} \varepsilon_{\max}=\left\{\begin{array}{cc} -\frac{1}{\lambda_{\min}}, & \mathrm{if}\;\lambda_{\min}<0\\ +\infty , & \mathrm{if}\;\lambda_{\min}\geq 0 \end{array}\right., \end{array}$$

*where $\lambda_{\min}$ is the minimal eigenvalue of $\Gamma_{A}\left[X\right]$.*

**Proof.**  Evidently, $\varepsilon_{\max}=\min\left\{s>0\;|\;\det\left(\exp_{A}\left(sX\right)\right)=0\right\}$. By (19), we have

(24) $$\begin{array}{c} \det\left(\exp_{A}\left(sX\right)\right)=\det\left(A\right)\det^{2}\left(I+s\Gamma_{A}\left[X\right]\right)=0\Leftrightarrow s=-\frac{1}{\lambda (\Gamma_{A}\left[X\right])}, \end{array}$$

where $\lambda (\Gamma_{A}\left[X\right])$ is the eigenvalue of $\Gamma_{A}\left[X\right]$. Thus, $\varepsilon_{\max}=\min\left\{-\frac{1}{\lambda (\Gamma_{A}\left[X\right])}>0\right\}$.    □

**Corollary** **3.** *The Wasserstein metric $g_{W}$ on SPD(n) is incomplete.*

Corollary 3 can be directly obtained from Hopf–Rinow theorem [22]. Theorem 5 and the next theorem help us to comprehend the size of $\left(SPD\left(n\right),g_{W}\right)$ from sense of each point.

**Figure 1:**
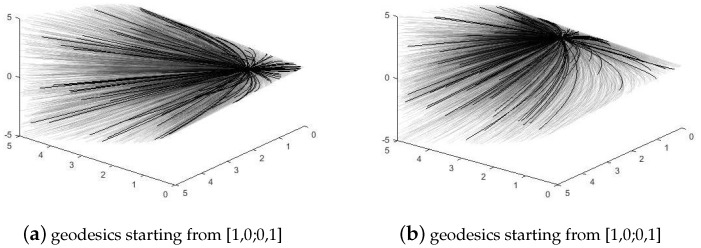
Geodesics of $g_{W}$ on SPD(2).

Figure 1 shows geodesics starting from different origins on SPD(2). From this group of pictures, we can observe the outline of the manifold and some behaviors of geodesics.

Using $\varepsilon_{\max}$, we can obtain the injectivity radius r(A),∀A∈SPD(n). Geometrically speaking, r(A) is the maximal radius of the ball in which $\exp_{A}$ is well-defined.

**Theorem** **6.** *The Wasserstein radius r(A):SPD(n)→(0,+∞) can be given by*

(25) $$\begin{array}{c} r\left(A\right)=\sqrt{\lambda_{\min}\left(A\right)}, \end{array}$$

*and the function r(A) is continuous.*

Proof of Theorem 6 can be found in Appendix C. Due to the geodesic convexity, the radius actually defines the Wasserstein distance of a point on SPD(n) to the ’boundary’ of the manifold. It also measures the degenerated degree of a positive-definite symmetric matrix by $\sqrt{\lambda_{\min}}$.

**Figure 2:**
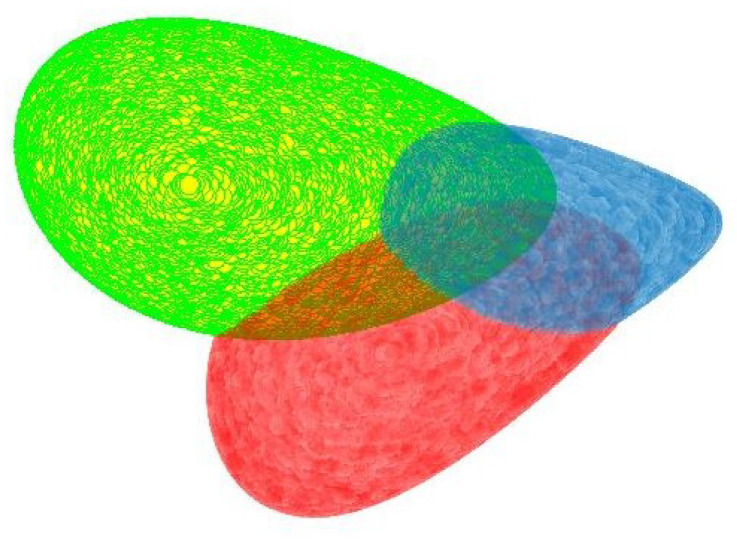
Three geodisical balls with the same radius.

Figure 2 shows three maximal geodisical balls with different centers on SPD(2). From the viewpoint of $R^{3}$, the three balls have different sizes in the sense of Euclidean distance, but on $\left(SPD\left(2\right),g_{W}\right)$, all of them have the same radius.

### 3.3. Connection

In this section, we will study the Riemannian connection of $\left(SPD\left(n\right),g_{W}\right)$, called a Wasserstein connection. The flatness of $\left(GL\left(n\right),g_{E}\right)$ and the structure of the Riemannian submersion will take a series of convenience to our work. During computation, we denote both tensor actions of $g_{W}$ on SPD(n) and $g_{E}$ on GL(n) by 〈·,·〉. Then, we denote the Euclidean connection as *D* and the Wasserstein connection as ∇.

The main idea to express the Wasserstein connection is to compute the horizontal term of the Euclidean covariant derivative of lifted vector fields. We shall prove:

**Lemma** **1.** *The Euclidean connection is a lift of the Wasserstein connection. For any smooth vector fields X and Y on SPD(n), and $\tilde{X}$ and $\tilde{Y}$ are their horizontal lifts, respectively, the following equation holds*

(26) $$\begin{array}{c} {d\sigma |}_{\tilde{A}}\left(D_{\tilde{X}}\tilde{Y}\right)=\nabla_{X}Y,\forall \tilde{A}\in GL\left(n\right). \end{array}$$

Proof of Lemma 1 can be found in Appendix D. This lemma holds for general Riemannian submersion. The reason we reprove it for the case is that we will need use some middle results of the proof later. Using Lemma 1, we can find a direct corollary, which is one of the essential results in this paper.

**Corollary** **4.** *The Wasserstein connection has an explicit expression:*

(27) $$\begin{array}{c} \nabla_{X}Y=dY\left(X\right)-\Gamma_{A}\left[X\right]A\Gamma_{A}\left[Y\right]-\Gamma_{A}\left[Y\right]A\Gamma_{A}\left[X\right], \end{array}$$

*where dY(X) is a Euclidean directional derivative.*

**Proof.**  From Lemma 1 and (A14) (in Appendix D), we have

(28) $$\begin{array}{cc} \nabla_{X}Y & {=d\sigma |}_{\tilde{A}}\left(D_{\tilde{X}}\tilde{Y}\right)={\tilde{A}}^{T}D_{\tilde{X}}\tilde{Y}+{\left(D_{\tilde{X}}\tilde{Y}\right)}^{T}\tilde{A}\\ \; & =dY\left(X\right)-\left(X\Gamma_{A}\left[Y\right]+\Gamma_{A}\left[Y\right]X\right)+A\Gamma_{A}\left[X\right]\Gamma_{A}\left[Y\right]+\Gamma_{A}\left[Y\right]\Gamma_{A}\left[X\right]A\\ \; & =dY\left(X\right)-\Gamma_{A}\left[X\right]A\Gamma_{A}\left[Y\right]-\Gamma_{A}\left[Y\right]A\Gamma_{A}\left[X\right]. \end{array}$$

The linearity, Leibnitz’s law and symmetry of Wasserstein connection are easily checked from the expression.    □

The vertical component of lifted covariant derivative of *Y* along *X* is a vector field in GL(n) whose value at $\tilde{A}$ is defined by

(29) $$\begin{array}{c} T_{\tilde{A}}\left(X,Y\right):=D_{\tilde{X}}\tilde{Y}-\tilde{\nabla_{X}Y}. \end{array}$$

We say $T_{\tilde{A}}$ is an A-tensor. The whole vector field is denoted as T(X,Y). By definition and previous results, we can obtain the expression of $T_{\tilde{A}}\left(X,Y\right)$.

**Proposition** **5.** *$T_{\tilde{A}}\left(\cdot ,\cdot \right)$ is a antisymmetric bilinear map: $T_{A}SPD\left(n\right)⊗T_{A}SPD\left(n\right)\to T_{\tilde{A}}GL\left(n\right)$, and it satisfies*

(30) $$\begin{array}{c} T_{\tilde{A}}\left(X,Y\right)=\tilde{A}\Gamma_{A}\left[\Pi \left[X,Y\right]\right]A. \end{array}$$

**Proof.**  Using (A12) (in Appendix D), (6) and (27), we have

(31) $$\begin{array}{cc} T_{\tilde{A}}\left(X,Y\right)=\; & \tilde{A}\left(\Gamma_{A}\left[dY\left(X\right)\right]-\Gamma_{A}\left[X\Gamma_{A}\left[Y\right]+\Gamma_{A}\left[Y\right]X\right]+\Gamma_{A}\left[X\right]\Gamma_{A}\left[Y\right]\right)\\ \; & -\tilde{A}\Gamma_{A}\left[dY\left(X\right)-\Gamma_{A}\left[X\right]A\Gamma_{A}\left[Y\right]-\Gamma_{A}\left[Y\right]A\Gamma_{A}\left[X\right]\right]\\ =\; & \tilde{A}\left(-\Gamma_{A}\left[A\Gamma_{A}\left[X\right]\Gamma_{A}\left[Y\right]\right]-\Gamma_{A}\left[\Gamma_{A}\left[Y\right]\Gamma_{A}\left[X\right]A\right]+\Gamma_{A}\left[X\right]\Gamma_{A}\left[Y\right]\right)\\ =\; & \tilde{A}\left(\Gamma_{A}\left[X\right]\Gamma_{A}\left[Y\right]-A\Gamma_{A}\left[\Gamma_{A}\left[X\right]\Gamma_{A}\left[Y\right]\right]-\Gamma_{A}\left[\Gamma_{A}\left[Y\right]\Gamma_{A}\left[X\right]\right]A\right)\\ =\; & \tilde{A}\Gamma_{A}\left[\Gamma_{A}\left[X\right]\Gamma_{A}\left[Y\right]-\Gamma_{A}\left[Y\right]\Gamma_{A}\left[X\right]\right]A\\ =\; & \tilde{A}\Gamma_{A}\left[\Pi \left[X,Y\right]\right]A, \end{array}$$

where (31) shows that $T_{\tilde{A}}\left(X,Y\right)$ depends only on $\tilde{A}$ and the vectors on $T_{A}SPD\left(n\right)$. The multi-linearity and $T_{\tilde{A}}\left(X,Y\right)=-T_{\tilde{A}}\left(Y,X\right)$ are easily checked.    □

Recalling (A14) in Appendix D, we also find that

(32) $$\begin{array}{c} \left[\tilde{X},\tilde{Y}\right]=\tilde{\left[X,Y\right]}+2T\left(X,Y\right). \end{array}$$

In the following parts, we will show the tensor $T\left(X,Y\right)$ plays a significant role for computing curvature.

### 3.4. Curvature

In this part, we tend to understand the curvature of $\left(SPD\left(n\right),g_{W}\right)$. Although there exists some relevant results giving abstract expressions for general cases, we obtain simpler expressions and derive the scalar curvature via a special basis.

#### 3.4.1. Riemannian Curvature Tensor

First, we derive the Riemannian curvature of $\left(SPD\left(n\right),g_{W}\right)$. We denote the Euclidean curvature on bundle (null entirely) as $\tilde{R}$, and the Wasserstein (Riemannian) curvature on $\left(SPD\left(n\right),g_{W}\right)$ as *R*.

**Theorem** **7.** *For any A∈SPD(n), and X,Y are smooth vector fields on SPD(n), the Wasserstein curvature tensor $R\left(X,Y,X,Y\right):={\langle R_{XY}X,Y\rangle }_{A}$ at A has an explicit expression*

(33) $$\begin{array}{c} R\left(X,Y,X,Y\right)=3\mathrm{tr}(\Gamma_{A}\left[X\right]A\Gamma_{A}\left[\Gamma_{A}\left[X\right]\Gamma_{A}\left[Y\right]-\Gamma_{A}\left[Y\right]\Gamma_{A}\left[X\right]\right]A\Gamma_{A}\left[Y\right]). \end{array}$$

Proof of Theorem 7 can be found in Appendix E. The expression

(34) $$\begin{array}{c} R\left(X,Y,X,Y\right)={∥T\left(X,Y\right)∥}^{2} \end{array}$$

has been derived before by other research group in similar way. However, here we use another way to calculate curvature tensor and find a more explicit expression, which is easier than expanding ${∥T\left(X,Y\right)∥}^{2}$ directly. In addition to that, from ${∥T\left(X,Y\right)∥}^{2}\geq 0$ and (A23) (in Appendix E), we can obtain the following corollary.

**Corollary** **5.** *$\left(SPD\left(n\right),g_{W}\right)$ has non-negative curvatures, namely*

(35) $$\begin{array}{c} R(X,Y,X,Y)\geq 0. \end{array}$$

By solving the Sylvester equation with Algorithm 1, we can simplify the expression. We give the sectional curvature *K* of the section span{X(A),Y(A)}

(36) $$\begin{array}{c} {K|}_{A}\left(X,Y\right)=\frac{R(X,Y,X,Y)}{\langle X,X\rangle \langle Y,Y\rangle -{\langle X,Y\rangle }^{2}}=12\frac{\mathrm{tr}(E_{X}\Lambda \Gamma_{\Lambda }\left[E_{X},E_{Y}\right]\Lambda E_{Y})}{\mathrm{tr}\left(E_{X}C_{X}\right)\mathrm{tr}\left(E_{Y}C_{Y}\right)-{\mathrm{tr}}^{2}\left(E_{X}C_{Y}\right)}, \end{array}$$

where we use the same donations as Algorithm 1. In particular, in diagonal cases, we obverse that the sectional curvature conforms to the inverse ratio law

(37) $$\begin{array}{c} {K|}_{k\Lambda }\left(X,Y\right)=\frac{1}{k}{K|}_{\Lambda }\left(X,Y\right),\forall k\in R-\left\{0\right\}. \end{array}$$

These results conform with our visualized views of $\left(SPD\left(n\right),g_{W}\right)$, as presented in Figure 1, where the manifold tends to be flat when *k* increases.

#### 3.4.2. Sectional Curvature

Now, we derive more explicit expressions for sectional curvature and scalar curvature. Conventionally, we only need to consider diagonal cases. Before that, we introduce a basis on Sym(n), which is the tangent space of SPD(n). Define $\left\{S^{p,q}\right\}$ as

(38) $$\begin{array}{c} S^{p,q}=\left[S_{ij}^{p,q}\right],S_{ij}^{p,q}=\delta_{i}^{p}\delta_{j}^{q}+\delta_{i}^{q}\delta_{j}^{p}, \end{array}$$

where the superscripts p,q marks the nonzero elements in $S^{p,q}$ and δ is the Kronecker delta. Apparently, $\left\{S^{p,q}\;|\;1\leq p\leq q\leq n\right\}$ forms a basis of Sym(n). For simplicity, we sometimes sign $S^{p,q},S^{r,t}$ with $S_{1},S_{2}$, respectively. In this way, we can express the curvature under this basis.

By direct calculation, we have

(39) $$\begin{array}{c} {\left(S_{1}S_{2}\right)}_{ij}=\sum_{k=1}^{n}S_{ik}^{p,q}S_{kj}^{r,t}=\delta_{i}^{p}\delta^{qt}\delta_{j}^{r}+\delta_{i}^{p}\delta^{qr}\delta_{j}^{t}+\delta_{i}^{q}\delta^{pt}\delta_{j}^{r}+\delta_{i}^{q}\delta^{pr}\delta_{j}^{t}. \end{array}$$

By Algorithm 1, we know that $E_{S}=\left(\frac{S_{ij}}{\lambda_{i}+\lambda j}\right)=\Gamma_{\Lambda }S,\forall S\in T_{\Lambda }SPD\left(n\right)$(Q=I in the decomposition of Λ). Note that the elements of $\Lambda ,S_{1},S_{2}$ are all positive; therefore, we have

(40) $$\begin{array}{c} E_{S_{1}}E_{S_{2}}\neq 0⟺S_{1}S_{2}\neq 0⟺\left\{p,q\right\}\cap \left\{r,t\right\}\neq \emptyset . \end{array}$$

According to the anti-symmetry of curvature tensor, the non-vanishing curvature means that {p,q}≠{r,t}. Moreover, by definition we know $S^{p,q}=S^{q,p},S^{r,t}=S^{t,r}$. Without loss of generality, we only need to consider the following particular case:

(41) $$\begin{array}{c} p=r,q\neq t. \end{array}$$

**Theorem** **8.** *For any diagonal matrix $\Lambda =\mathrm{diag}\left(\lambda_{1},\cdots ,\lambda_{n}\right)\in SPD\left(n\right)$, where $\lambda_{1}\leq \lambda_{2}\leq \cdots \leq \lambda_{n}$, Wasserstein sectional curvature satisfies*

(42) $$\begin{array}{c} {K|}_{\Lambda }\left(S_{1},S_{2}\right)=\frac{3\left(1+\delta^{pq}\right)\left(1+\delta^{pt}\right)\lambda_{q}\lambda_{t}}{\left(\lambda_{p}+\lambda_{q}\right)\left(\lambda_{p}+\lambda_{t}\right)\left(\lambda_{q}+\lambda_{t}\right)}, \end{array}$$

*where $S_{1}=S^{p,q},S_{2}=S^{r,t},p=r,q\neq t$.*

Proof of Theorem 8 can be found in Appendix F. With the above expansion for sectional curvatures, we can easily find that sectional curvature can be controlled by the secondly minimal eigenvalue, which implies that the curvature will seldom explode even on a domain almost degenerated. Only when the matrices degenerate at over two dimensions will the curvatures be very large. This phenomenon ensures the curvature information makes sense in most applications. Some examples for this phenomenon can be observed later.

#### 3.4.3. Scalar Curvature

In the last part of this section, we calculate the scalar curvature directly.

**Theorem** **9.** *For any A∈SPD(A), its scalar curvature ρ(A) is*

(43) $$\begin{array}{c} \rho \left(A\right)=3\mathrm{tr}(U\Lambda \left(U+U^{T}\right)+\left(U+U^{T}\right)\Lambda U+\left(U+U^{T}\right)\Lambda U\Lambda \left(U+U^{T}\right)), \end{array}$$

*where the diagonal matrix $\Lambda =\mathrm{diag}(\lambda_{1},\cdots ,\lambda_{n})$ is orthogonal similar to A, and $U={\left(\frac{1}{\lambda_{i}+\lambda_{j}}\right)}_{i<j}$.*

**Figure 3:**
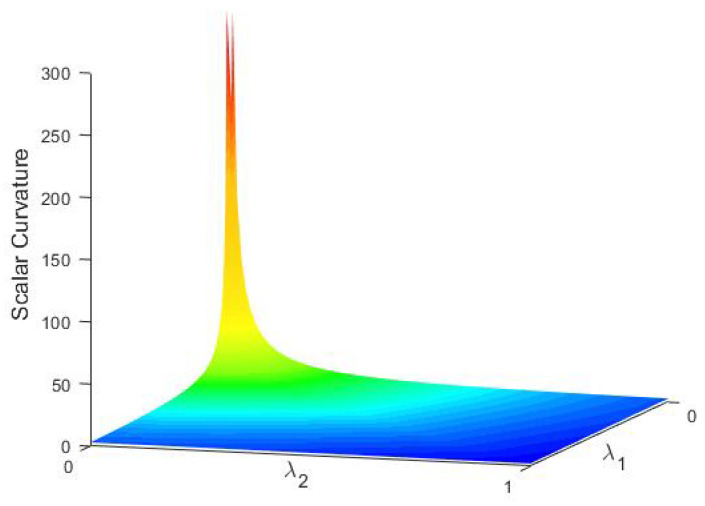
Scalar curvatures on *SPD*(2).

Proof of Theorem 9 can be found in Appendix G. Figure 3 presents some examples for scalar curvatures on $\left(SPD\left(2\right),g_{W}\right)$, which shows our argument in the last part of Section 3.4.2.

## 4. Point Cloud Denoising

Denoising or outlier removal is a fundamental step of point cloud preprocessing since real-world data are often polluted by noise. There are immense literature in point cloud denoising and widely used algorithms packed as inline functions of softwares. For example, PCL [16] is a popular platform for point cloud processing, which collects four denoising schemes. However, these methods fail to give satisfactory performance when point clouds are polluted by high density noise. To solve this problem, we consider both the statistical and geometrical structure of data and design a new algorithm.

The idea is that by embedding the original point cloud from Euclidean space into SPD(n), the Wasserstein scalar curvature gives essential information about noise and true data. Therefore, our new algorithm mainly contains two steps: First, we give the desired embedding by fitting a Gaussian distribution locally at each point. Then, we identify noise by looking at the histogram of the Wasserstein scalar curvature. Due to the flatness of the space of noise, it is reasonable to classify points with small curvature to be noise. The threshold is set to be the first local minimum of the histogram. We call this new scheme adaptive Wasserstein curvature denoising (AWCD).

In the following, we introduce two traditional denoising methods called radius outlier removal (ROR) and statistical outlier removal (SOR). Then, we explain details about AWCD. Additionally, we carry out experiments using different datasets, with a comparison to two classical methods. From the experimental results, AWCD presents better performance regardless of the data size and the density of noise. We also give a time complexity analysis for each denoising algorithm. The results show that AWCD is as efficient as other classical methods. Thus, it is applicable in many practical tasks.

### 4.1. Radius Outlier Removal

In Radius Outlier Removal, called ROR (seeing Algorithm 2), points are clustered into two categories according to their local density, i.e., points with low density tend to be recognized as noise, whereas points with high density are recognized as true data. ROR requires two parameters: a preset parameter *d* as the radius for local neighborhoods and α as the least number of points in each neighborhood.
**Algorithm 2** Radius Outlier Removal.**Input:** initial point cloud $D_{0}$, parameters *d*, α**Output:** cleaned point cloud $D_{1}$1:search *d*-radius neighborhood $N_{i}$ for each point $P_{i}$, where
$$N_{i}=\left\{N_{ij}\in D_{0}|∥N_{ij}-P_{i}∥\leq d\right\};$$2:**if** number of neighbors $|N_{i}|\geq \alpha$ **then** put $P_{i}$ into $D_{1}$;3:**return**$D_{1}$.

**Figure 4:**
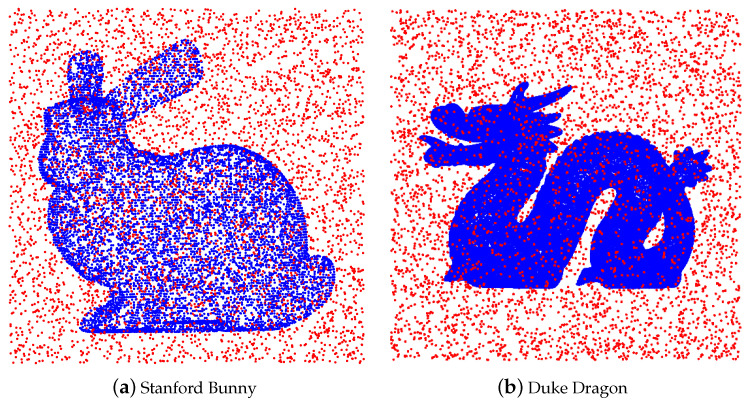
Stanford Bunny with uniform noise.

As an illustration, we add uniform noise to the Stanford Bunny with 10,000 points (see Figure 4).

**Figure 5:**
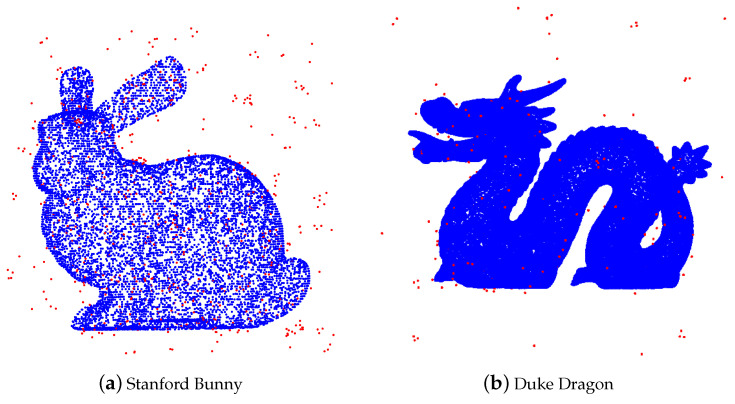
Cleaned point cloud by ROR.

Then, we apply ROR to denoise the polluted point cloud. The result is shown in Figure 5. From a visual observation, ROR preserves almost all true points but fails to recognize a small portion of noise at any area.

In fact, from a series of repetitive experiments we find that ROR is sensitive to the choice of manual parameters. A small radius will make ROR inefficient, while a large radius will wrongly recognize true points as noise. One of the disadvantages of ROR is that there exists no universal method to determine the best parameters. Further, since ROR uses the kernel method to find the undetermined closest neighbors, the time complexity can reach to $O(n^{2})$ where *n* is the number of points. Thus, in practice, it is often difficult to make a trade-off between efficiency and effect of ROR.

### 4.2. Statistical Outlier Removal

Compared to ROR, Statistical Outlier Removal (SOR) considers more detailed local structures than density does. SOR showed in Algorithm 3 is one of the most popular methods to preprocess point clouds due to its efficiency when dealing with low density noise. However, SOR gives worse performance than ROR when the noise is of high density. The main idea of SOR comes from one-sigma law from classical statistics [23]. An outlier is believed to be far from the center of its k-nearest neighborhood. Conversely, a true point should lie in a confidence area of its neighborhoods. Let Φ be a *d*-variate Gaussian distribution with expectation μ and covariance Σ, and let *P* be a fixed point in $R^{d}$. Then, Φ induces a Gaussian distribution on the line $\left\{\mu +tv_{P}|t\in R\right\}$ where $v_{P}=\frac{P-\mu }{∥P-\mu ∥}$. In fact, we write the eigendecomposition of Σ as $\Sigma =E\mathrm{diag}\left(\sigma_{1}^{2},\cdots ,\sigma_{d}^{2}\right)E^{T}$, where $E=[e_{1},\cdots ,e_{d}]$ is an orthogonal matrix. If we write

(44) $$v_{P}=\sum_{i=1}^{d}\lambda_{i}e_{i},$$

the projected Gaussian distribution in direction $v_{P}$ has null expectation and variance $\sum_{i=1}^{d}\lambda_{i}^{2}\sigma_{i}^{2}$. According to one-sigma law, we say *P* is in the confidence area of Φ if

(45) $${∥P-\mu ∥}^{2}\leq \sum_{i=1}^{d}\lambda_{i}^{2}\sigma_{i}^{2},$$

which is equivalent to

(46) $${\left(P-\mu \right)}^{T}\Sigma \left(P-\mu \right)\geq {∥P-\mu ∥}^{4}.$$

This inequality is a generalization of one-sigma law in high dimensions.
**Algorithm 3** Statistical Outlier Removal.**Input:** initial point cloud $D_{0}$, parameter *k***Output:** cleaned point cloud $D_{1}$1:search kNN $N_{i}$ for each point $P_{i}$;2:compute local mean and local covariance
$$\mu_{i}=\frac{1}{k}\sum_{j=1}^{k}N_{ij},\;\Sigma_{i}=\frac{1}{k-1}\sum_{j=1}^{k}{\left(N_{ij}-\mu_{i}\right)}^{T}\left(N_{ij}-\mu_{i}\right);$$3:**if**${\left(P_{i}-\mu_{i}\right)}^{T}\Sigma_{i}\left(P_{i}-\mu_{i}\right)\geq {∥P_{i}-\mu_{i}∥}^{4}$**then** put $P_{i}$ into $D_{1}$;4:**return**$D_{1}$.

**Figure 6:**
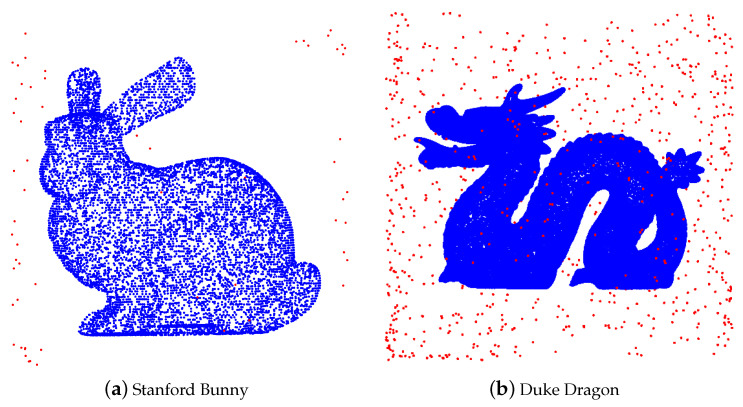
Cleaned point cloud by SOR.

Thus, SOR consists of three steps: first we search the k-nearest neighbors (kNN) for every point. Then, we compute the empirical mean and covariance under the assumption of Gaussian distribution for each neighborhood. Finally, true points are identified using (46). SOR requires a single parameter *k* for kNN. Again, as an illustration, we use the data in Figure 4. After SOR, the result is shown in Figure 6.

We use KD-tree in kNN search. Thus, the time complexity is known as O(knlogn) where *k* is the number of neighbors and *n* is the number of points. The remaining steps are finished in O(n) time. Therefore, the total time complexity is O(knlogn).

### 4.3. Adaptive Wasserstein Curvature Denoising

Note that the key step in SOR is to compute the local covariance, which is a positive-definite matrix. Motivated by the idea of SOR, we extract the covariance matrix at each point, which is equivalent to embed the original point cloud into SPD(n). From an intuitive perspective, since the true data presents a particular pattern, the covariance matrices should have a large Wasserstein curvature. Conversely, for noise, the covariance matrices form a flat region. Hence, AWCD is based on a principal hypothesis that the Wasserstein curvature of true data is larger than noise.

**Figure 7:**
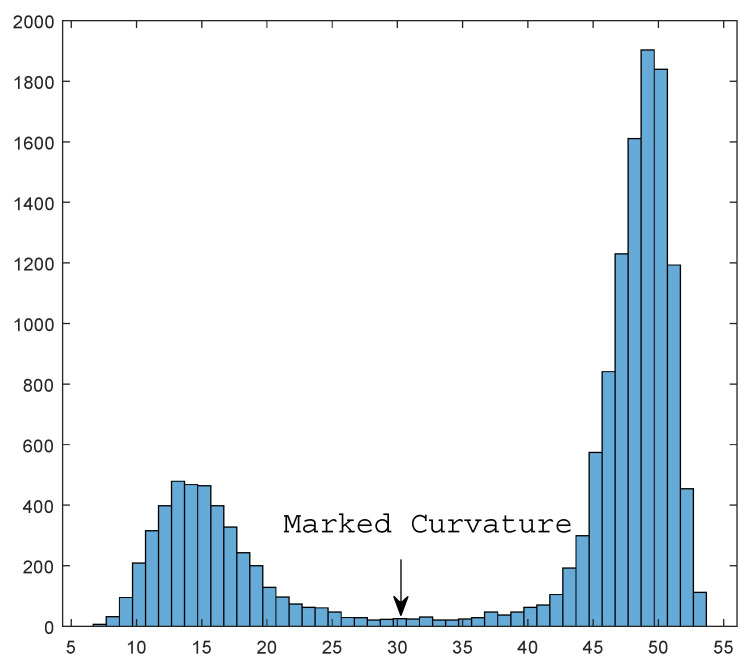
Histogram of the Wasserstein scalar curvature; *x*-axis: scalar curvature and *y*-axis: point number.

Under such a hypothesis, what we need to do is to set a threshold to pick out points with a small Wasserstein curvature. To do so, we gather all information in a histogram counting the number of points of different curvature. By the continuity of curvature function, true data and noise will form two different ’hills’. Figure 7 shows an example for the histogram.

The phase change happens at the borderline of two hills, i.e., we seek to find the second minimal value of the histogram. In Figure 7, the critical value is annotated as ’marked curvature’. In this way, we do not need to set the threshold manually and, instead, achieve an adaptive selection process. Algorithm 4 shows the processing of this adaptive denoising via wasserstein curvature.
**Algorithm 4** Adaptive Wasserstein Curvature Denoising.**Input:** initial point cloud $D_{0}$, parameter *k***Output:** cleaned point cloud $D_{1}$1:search kNN neighbors $N_{i}$ for each point $P_{i}$;2:compute local mean and local covariance as before;3:compute Wasserstein curvature ρ(Σ) as (43);4:construct curvature histogram and determine the marked curvature $\rho_{0}$;5:**if**$\rho \left(\Sigma_{i}\right)\geq \rho_{0}$**then** put $P_{i}$ into $D_{1}$;6:**return**$D_{1}$.

**Figure 8:**
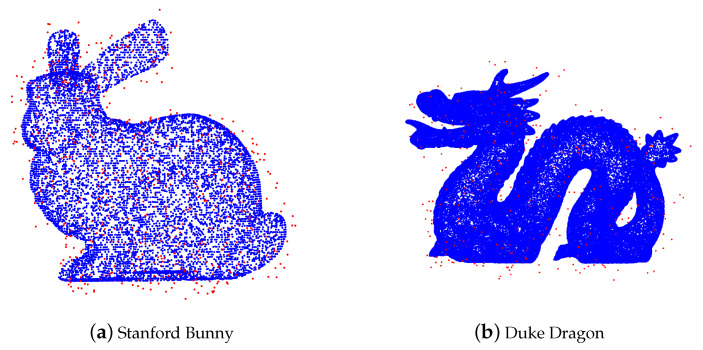
Cleaned point cloud by AWCD.

We use the same example as in Figure 4. The performance of AWCD is shown in Figure 8.

In this example, AWCD removes almost all noise far from Stanford Bunny, and remains almost all true data. The only problem is that a small portion of noise lying on the dragon cause the false positiveness and some true data located on the flat part are wrongly removed.

Since the main step in AWCD is also kNN, the time complexity is the same as SOR, which is O(knlogn). Therefore, AWCD is applicable in practice. It is remarkable that AWCD is effective for data with dense noise and robust to the unique parameter *k*.

### 4.4. Experiments

We use ROR, SOR and AWCD to denoise polluted data sets with noise of different levels of densities. The point clouds are from the Stanford 3D scanning repository, including Stanford Bunny, Duke Dragon, Armadillo, Lucy and Happy Buddha. For each data set, we add noise and record its signal-noise ratio (SNR). To show the influence of data size, we downsample the original data sets of different scales.

We adopt three criteria to measure the performance of the algorithms, including true positive rate (TPR), false positive rate (FPR) and signal-noise rate growing (SNRG). TPR describes the accuracy to preserve true points from unpolluted data sets. FPR describes the success rate to remove noisy points. SNRG explicates the promotion of SNR after processing. For any polluted point cloud $D_{0}=D\cup N$, where *D* is the points set of true data and *N* is the set of noise. We obtain the cleaned point cloud $D_{1}$ after the denoising algorithms. Then, the computation of these measurements are

(47) $$\begin{array}{cc} \; & \mathrm{TPR}=\frac{|D_{1}\cap D|}{\left|D\right|},\\ \; & \mathrm{FPR}=1-\frac{|D_{1}\cap N|}{\left|N\right|},\\ \; & \mathrm{SNRG}=\frac{|D_{1}\cap D|}{|D_{1}\cap N|}\cdot \frac{\left|N\right|}{\left|D\right|}-1, \end{array}$$

where |·| denotes the cardinality or size of a finite set. Intuitively, higher TPR, SNRG and lower FPR mean better performance of an algorithm. The experimental results are shown in Table A1 in Appendix I. In each experiment, we highlight the lowest FPR, the highest TPR and the SNRG over 99%. Table A1 shows the superiority of AWCD to ROR and SOR. In general, AWCD can remove almost all noise and meanwhile preserves the true data, except for Armadillo.

## 5. Edge Detection

In this part, we attempt to apply the Wasserstein curvatures to detect the edges of images with noises. This application follows the idea that the edge parts contain more local information while the relatively flat parts tend to be regarded locally as white noise. Hence, the Wasserstein curvatures have natural advantages to depict the local information. This leads to the following Wasserstein sectional curvature edge detection (WSCED) of Algorithm 5.
**Algorithm 5** Wasserstein sectional curvature edge detection.**Input:** initial grayscale $F_{0}$ with pixels of n×m, parameter *k***Output:** edge figure $F_{e}$1:search kNN $N_{ij}$ for each point $P_{ij}$;2:compute every local covariance $\Sigma_{ij}$ to obtain the covariance image CI, which is a (n−2k)×(m−2k) matrix constructed by matrixes $\Sigma_{ij}$;3:determine the section $\sigma_{ij}:=X_{ij}\wedge Y_{ij}$ for every point $\Sigma_{ij}$ on CI by computing tangent vectors $X_{ij}=\left(\Sigma_{(i+1)j}-\Sigma_{(i-1)j}\right),Y_{ij}=\left(\Sigma_{i(j+1)}-\Sigma_{i(j-1)}\right)$;4:compute the Wasserstein sectional curvature for every ${Kw|}_{\Sigma_{ij}}\left(\sigma_{ij}\right)$ with (36) to obtain the curvature image $F_{e}$, which is a (n−2k)×(m−2k) real matrix;5:**return**$F_{e}$.

Similar to what we have done in the last section, the first step of WSCED is computing the local mean and variance after kNN, which can be regarded as a two-dimensional embedding from a image into SPD(n). Every pixel coordinate (i,j) determines a local covariance matrix $\Sigma_{ij}$. In the second step, we compute the sectional curvature for every $\Sigma_{ij}$. The chosen section $X_{ij}\wedge Y_{ij}$ is determined by two difference vectors along *x*-axis and *y*-axis,

(48) $$\begin{array}{cc} \; & X_{ij}:=\Sigma_{(i+1)j}-\Sigma_{(i-1)j},\\ \; & Y_{ij}:=\Sigma_{i(j+1)}-\Sigma_{i(j-1)}. \end{array}$$

According to (36), we obtain the chosen curvature ${KI}_{ij}=K_{w}{|}_{\Sigma_{ij}}\left(X_{ij},Y_{ij}\right)$ on $\Sigma_{ij}$. Then, we obtain a curvature image KI. Finally, with some appropriate image transformation, we can detect edges on KI.

**Figure 9:**
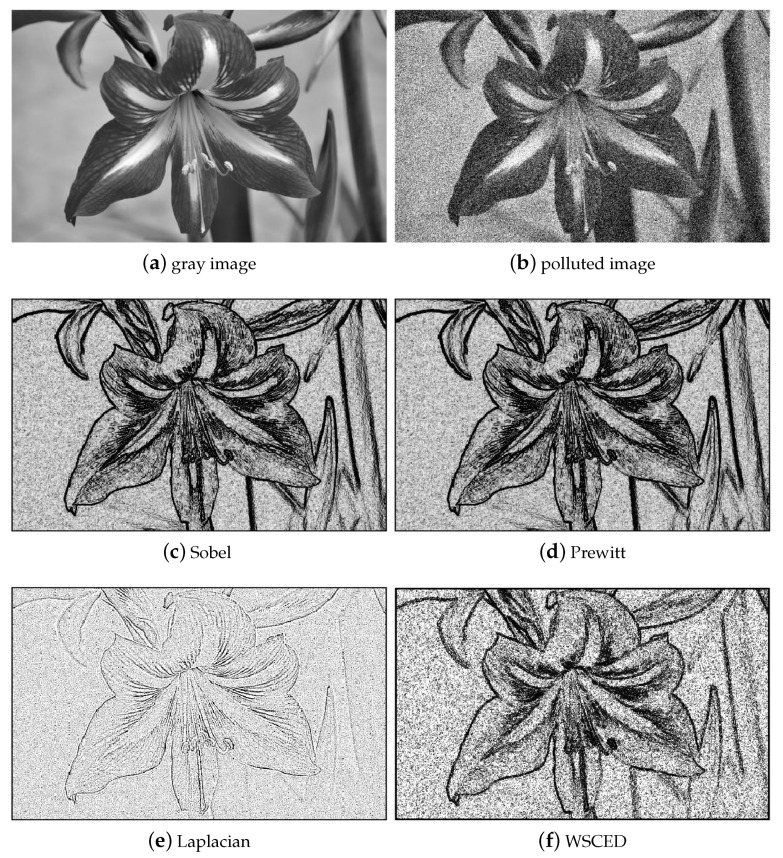
An example to show different edge detection algorithms on the flower image.

**Figure A1:**
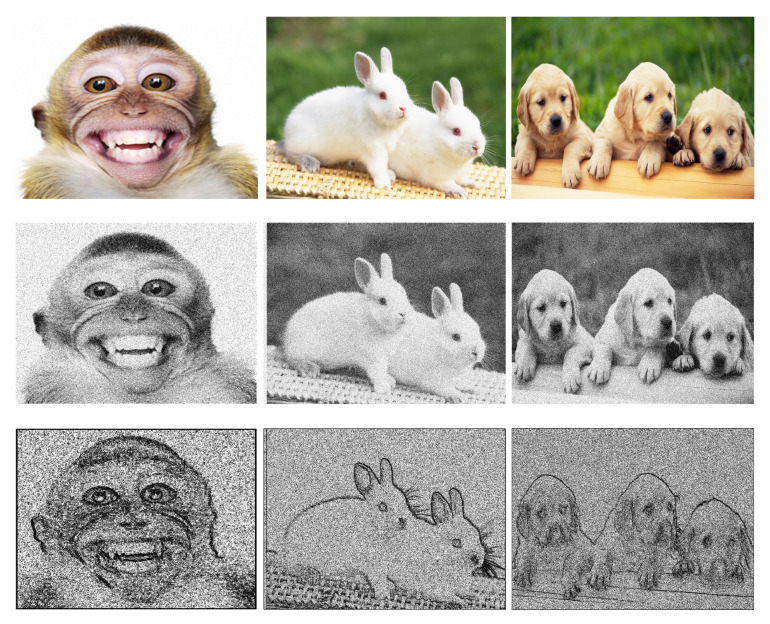
WSCED on different polluted images. The first row shows the original RGB images. The second row shows images with uniform noises. The third row shows edge images after WSCED.

In simulations, we compare WSCED to traditional edge detecting filters, including Sobel, Prewitt and Laplacian [24]. We tend to detect edges for images with noises in high density. From Figure 9, we find that WSCED approaches the same outcome as Sobel and Prewitt filters, which implies the potential connection between Wasserstein curvature and edges. This result also shows the robustness of WSCED to noises. We present more effects of digital experiments in Figure A1 in Appendix H.

## 6. Conclusions and Future Work

In this paper, we studied the geometric characteristics of $\left(SPD\left(n\right),g_{W}\right)$, including geodesics, the connection, Jacobi fields and curvatures. Compared with the existing results, our results are simpler in form and more suitable for computation. Based on these results, we designed novel algorithms for point cloud denoising and image edge detection. Numerical experiments showed that these geometry-based methods were valid for applications. From both a theoretical and practical prospective, we gained a more comprehensive understanding regarding the Wasserstein geometry on SPD(n), which shows that the Wasserstein metric has both deep application potential and mathematical elegance.

In our future work, on the one hand, we aim to study Wasserstein geometry on other matrix manifolds, such as the Stiefel manifold [25], Grassman manifold [26] and some complex matrix manifolds [27]. On the other hand, we would like to generalize geometry-based methods to solve more problems in image, signal processing [28] and data science.

## Data Availability

In this paper, we obtain all of our point clouds data from the Large Geometric Models Archive of Georgia Tech at https://search.crossref.org/funding (on 5 April 2021). The images were gained from the open resources from Google.

## Appendix A. Properties of Γ_*A*_ [X]

Here, we list some basic properties, which will be used frequently in the discussions of this paper.

**Proposition** **A1.**
*For any A,B∈SPD(n),X,Y∈M(n),Q∈O(n),k∈R, the following equations hold:*

1. *$\Gamma_{A}\left[X+kY\right]=\Gamma_{A}\left[X\right]+k\Gamma_{A}\left[Y\right]$;*
2. *$\Gamma_{kA}\left[X\right]=\frac{1}{k}\Gamma_{A}\left[X\right]$;*
3. *$\Gamma_{A+B}\left[X\right]=\Gamma_{A}\left[X\right]-\Gamma_{A}\left[B\Gamma_{A+B}\left[X\right]+\Gamma_{A+B}\left[X\right]B\right],A+B\in SPD\left(n\right)$;*
4. *$\Gamma_{A}\left[AX\right]=A\Gamma_{A}\left[X\right],\Gamma_{A}\left[XA\right]=\Gamma_{A}\left[X\right]A$;*
5. *$\Gamma_{A^{-1}}\left[X\right]=\Gamma_{A}\left[AXA\right]=A\Gamma_{A}\left[X\right]A$;*
6. *$\Gamma_{QAQ^{-1}}\left[QXQ^{-1}\right]=Q\Gamma_{A}\left[X\right]Q^{-1}$.*

Proposition A1 will determine the geometry on $\left(SPD\left(n\right),g_{W}\right)$ and be involved into every calculation repetitively and alternatively. We omit the proof since these properties are easily checked.

## Appendix B. Proof of Theorem 1

To prove Theorem 1, we shall give two lemmas first.

**Lemma** **A1.**
*For any ${\tilde{A}}_{1},{\tilde{A}}_{2}\in GL\left(n\right)$, $l\left(t\right)=t{\tilde{A}}_{2}+\left(1-t\right){\tilde{A}}_{1},t\in \left[0,1\right]$. $\dot{l}\left(t\right)$ is horizontal if and only if det(l(t))>0, and $\dot{l}\left(0\right)$ is horizontal at $T_{{\tilde{A}}_{1}}GL\left(n\right)$.*

**Proof.** 

(A1) $$\begin{array}{cc} \; & \dot{l}\left(0\right)={\tilde{A}}_{2}-{\tilde{A}}_{1}\;\mathrm{is}\;\mathrm{horizontal}\;\mathrm{at}\;T_{{\tilde{A}}_{1}}GL\left(n\right)\\ \Leftrightarrow \; & {\tilde{A}}_{1}^{-1}\left({\tilde{A}}_{2}-{\tilde{A}}_{1}\right)\in Sym\left(n\right)\\ \Leftrightarrow \; & {\tilde{A}}_{1}^{-1}{\tilde{A}}_{2}\in Sym\left(n\right)\\ \Leftrightarrow \; & {\tilde{A}}_{2}^{-1}{\tilde{A}}_{1}\in Sym\left(n\right)\\ \Leftrightarrow \; & {\tilde{A}}_{2}^{-1}\left({\tilde{A}}_{2}-{\tilde{A}}_{1}\right)\in Sym\left(n\right)\\ \Leftrightarrow \; & \dot{l}\left(1\right)\;\mathrm{is}\;\mathrm{also}\;\mathrm{horizontal}\;\mathrm{at}\;T_{{\tilde{A}}_{2}}GL\left(n\right). \end{array}$$

For any s∈(0,1) that satisfies det(l(s))≠0, we constrict the line segment by $l_{s}\left(t\right):=l\left(st\right)$, and the lemma can be induced by a horizontal of ${\dot{l}}_{s}\left(1\right)$. □

Lemma A1 ensures that a line segment is horizontal if its initial vector is horizontal, which brings quite convenience for the following proofs of certain results.

**Lemma** **A2.**
*For any $A_{1},A_{2}\in SPD\left(n\right)$, there exists $P=A_{1}^{-\frac{1}{2}}{\left(A_{1}A_{2}\right)}^{\frac{1}{2}}A_{2}^{-\frac{1}{2}}\in O\left(n\right)$ such that $\tilde{\gamma }\left(t\right)=tPA_{2}^{\frac{1}{2}}+\left(1-t\right)A_{1}^{\frac{1}{2}}$. When $\det(\tilde{\gamma }\left(t\right))>0$, $\dot{\tilde{\gamma }}\left(t\right)$ keeps horizontal at $T_{\tilde{\gamma }\left(t\right)}GL\left(n\right)$.*

**Proof.** First, we have

(A2) $$\begin{array}{cc} P^{T}P & =A_{2}^{-\frac{1}{2}}{\left(A_{2}A_{1}\right)}^{\frac{1}{2}}A_{1}^{-1}{\left(A_{1}A_{2}\right)}^{\frac{1}{2}}A_{2}^{-\frac{1}{2}}\\ \; & =A_{2}^{-\frac{1}{2}}A_{1}^{-1}{\left(A_{1}A_{2}\right)}^{\frac{1}{2}}{\left(A_{1}A_{2}\right)}^{\frac{1}{2}}A_{2}^{-\frac{1}{2}}\\ \; & =I. \end{array}$$

Then, we only need to check that $A_{1}^{-\frac{1}{2}}\dot{\tilde{\gamma }}\left(0\right)\in Sym\left(n\right)$. In fact, we have

(A3) $$\begin{array}{cc} A_{1}^{-\frac{1}{2}}\dot{\tilde{\gamma }}\left(0\right) & =A_{1}^{-1}{\left(A_{1}A_{2}\right)}^{\frac{1}{2}}-I\\ \; & =A_{1}^{-\frac{1}{2}}A_{1}^{-\frac{1}{2}}{\left(A_{1}A_{2}\right)}^{\frac{1}{2}}A_{1}^{\frac{1}{2}}A_{1}^{-\frac{1}{2}}-I\\ \; & =A_{1}^{-\frac{1}{2}}{\left(A_{1}^{\frac{1}{2}}A_{2}A_{1}^{\frac{1}{2}}\right)}^{\frac{1}{2}}A_{1}^{-\frac{1}{2}}-I\in Sym\left(n\right), \end{array}$$

which proves the lemma. □

**Remark** **A1.**
*Finding the orthogonal matrix P is equivalent to solving a Riccati equation. One can see [29] for details.*

To prove Theorem 1, the last step is to clarify the non-degeneration.

**Proof** **.** (Theorem 1) Due to the symmetry from (10), it suffices to prove the diagonal case $A_{1}=\Lambda$. We shall prove $\det\left(\tilde{\gamma }\left(t\right)\right)>0$, ∀t∈(0,1). We have

(A4) $$\begin{array}{cc} \det[\tilde{\gamma }\left(t\right)] & =\det\left[t\Lambda^{-\frac{1}{2}}{\left(\Lambda A_{2}\right)}^{\frac{1}{2}}+\left(1-t\right)\Lambda^{\frac{1}{2}}\right]\\ \; & ={\left(1-t\right)}^{n}\det\left[I+\frac{t}{\left(1-t\right)}\Lambda^{-\frac{1}{2}}{\left(\Lambda A_{2}\right)}^{-\frac{1}{2}}\Lambda^{-\frac{1}{2}}\right]\det\left[\Lambda^{\frac{1}{2}}\right]. \end{array}$$

Since $\Lambda^{-\frac{1}{2}}{\left(\Lambda A_{2}\right)}^{\frac{1}{2}}\Lambda^{-\frac{1}{2}}$ is congruent to ${\left(\Lambda A_{2}\right)}^{\frac{1}{2}}$, its eigenvalues are all positive. Thus, $\det[\tilde{\gamma }\left(t\right)]>0$ holds for all t∈(0,1). Combining with Lemmas A1 and A2, the proof is done. □

## Appendix C. Proof of Theorem 6

**Proof.** Again, with (10), we have r(A)=r(Λ) where $A=Q\Lambda Q^{T}$. Thus, we still focus on r(Λ). Following the definition and discussions around (23),

(A5) $$\begin{array}{cc} r(\Lambda ) & =\inf\{\epsilon \left(V\right)\;|\;V\in T_{\Lambda }SPD\left(n\right),∥V{∥}_{g_{W}}=1\}\\ \; & =\inf\{∥V{∥}_{g_{W}}\;|\;V\in T_{\Lambda }SPD\left(n\right),\det\left(I+\Gamma_{\Lambda }\left[V\right]\right)=0\}, \end{array}$$

in which ${∥\cdot ∥}_{g_{W}}$ is the inner product reduced by $g_{W}$. Then, we have

(A6) $$\begin{array}{c} 2r^{2}\left(\Lambda \right)=\inf\left\{\mathrm{tr}\left(V\Gamma_{\Lambda }\left[V\right]\right)\;|\;\Gamma_{\Lambda }\left[V\right]\;\mathrm{has}\;\mathrm{eigenvalue}\;\eta_{k}=-1\right\}. \end{array}$$

Denote $\left\{\lambda_{i}>0\right\}$ as eigenvalues of Λ, and $\eta_{j}$ as eigenvalues of $\Gamma_{\Lambda }\left[V\right]$. Let $x_{j}$ be eigenvectors corresponding to $\eta_{j}$ such that $\left\{x_{j}\right\}$ forms an orthogonal basis of $R^{n}$. By direct calculation, we find

(A7) $$\begin{array}{cc} \mathrm{tr}(V\Gamma_{\Lambda }\left[V\right]) & =\;\sum_{i=1}^{n}x_{i}^{T}V\Gamma_{\Lambda }\left[V\right]x_{i}=\sum_{i=1}^{n}\eta_{i}x_{i}^{T}Vx_{i}\\ \; & =\;\sum_{i=1}^{n}\eta_{i}x_{i}^{T}\left(\Lambda \Gamma_{\Lambda }\left[V\right]+\Gamma_{\Lambda }\left[V\right]\Lambda \right)x_{i}\\ \; & =\;\sum_{i=1}^{n}\left(\eta_{i}{\left(\Gamma_{\Lambda }\left[V\right]x_{i}\right)}^{T}\Lambda x_{i}+\eta_{i}x_{i}^{T}\Lambda \Gamma_{\Lambda }\left[V\right]x_{i}\right)\\ \; & \;=2\sum_{i=1}^{n}\eta_{i}^{2}x_{i}^{T}\Lambda x_{i}\geq 2x_{k}^{T}\Lambda x_{k}, \end{array}$$

where we used the facts that Γ is positive-definite and $\eta_{k}=-1$. The equality $\mathrm{tr}\left(V\Gamma_{\Lambda }\left[V\right]\right)=x_{k}^{T}\Lambda x_{k}$ holds if and only if $\eta_{j}=0,\forall j\neq k$. In these cases, by Algorithm 1, we have

(A8) $$\begin{array}{c} \frac{V_{ij}}{\lambda_{i}+\lambda_{j}}={\left(\Gamma_{\Lambda }\left[V\right]\right)}_{ij}=-\delta_{ik}\delta_{jk}, \end{array}$$

then we obtain that

(A9) $$\begin{array}{c} V_{ij}=-2\delta_{ik}\delta_{ik}\lambda_{k},{\left(x_{k}\right)}_{i}=\delta_{ki}. \end{array}$$

Thus,

(A10) $$\begin{array}{c} \mathrm{tr}\left(V\Gamma_{\Lambda }\left[V\right]\right)=2x_{k}^{T}\Lambda x_{k}=2\lambda_{k}. \end{array}$$

In particular, we obtain $2\lambda_{\min}$ as $\min\{\mathrm{tr}\left(V\Gamma_{\Lambda }\left[V\right]\right)\}$, when $\Lambda_{k}=\lambda_{\min}$ and $V_{ij}=-2\delta_{ik}\delta_{jk}\lambda_{\min}$. The tangent vector *V* is called the speed-degenerated direction. The function $\lambda_{\min}\left(A\right)$ is certainly continuous, hence r(A) is also continuous. □

## Appendix D. Proof of Lemma 1

Before proving Lemma 1, we shall prove a key lemma, which points the relation between the Lie-brackets on the total space and base space.

**Lemma** **A3.**
*The horizontal lift of vector fields commutes with Lie-brackets,*

(A11)
$$\begin{array}{c} {d\sigma |}_{\tilde{A}}\left[\tilde{X},\tilde{Y}\right]=\left[X,Y\right],\forall \tilde{A}\in GL\left(n\right). \end{array}$$

**Proof** **.** (Lemma A3) On the flat manifold $\left(GL\left(n\right),g_{E}\right)$, the connection equals to the usual directional derivative in the Euclidean space. Therefore, for vector fields X,Y on SPD(n), we have

(A12) $$\begin{array}{cc} D_{\tilde{X}}\tilde{Y} & =\lim_{t\to 0}\frac{1}{t}\left(\tilde{Y}{{|}_{\tilde{A}+\tilde{X}t}-\tilde{Y}|}_{\tilde{A}}\right)\\ \; & =\lim_{t\to 0}\frac{1}{t}\left(\left(\tilde{A}+\tilde{X}t\right)\Gamma_{A+Xt}\left[Y{|}_{A+Xt}\right]-\tilde{A}\Gamma_{A}\left[Y{|}_{A}\right]\right)\\ \; & =\lim_{t\to 0}\frac{\tilde{A}}{t}\left(\Gamma_{A+Xt}\left[Y{|}_{A+Xt}\right]-\Gamma_{A}\left[Y{|}_{A}\right]\right)+\tilde{X}\Gamma_{A}\left[Y\right]\\ \; & =\lim_{t\to 0}\frac{\tilde{A}}{t}\left(\Gamma_{A+Xt}\left[Y{|}_{A+Xt}\right]-\Gamma_{A}\left[Y{|}_{A}\right]\right)+\tilde{A}\Gamma_{A}\left[X\right]\Gamma_{A}\left[Y\right]\\ \; & =\tilde{A}\left(\Gamma_{A}\left[dY\left(X\right)\right]-\Gamma_{A}\left[X\Gamma_{A}\left[Y\right]+\Gamma_{A}\left[Y\right]X\right]+\Gamma_{A}\left[X\right]\Gamma_{A}\left[Y\right]\right). \end{array}$$

With $\Pi_{A}\left[X,Y\right]:=\Gamma_{A}\left[X\right]\Gamma_{A}\left[Y\right]-\Gamma_{A}\left[Y\right]\Gamma_{A}\left[X\right]$ into (2), we obtain

(A13) $$\begin{array}{c} \Gamma_{A}\left[X\Gamma_{A}\left[Y\right]+\Gamma_{A}\left[Y\right]X-Y\Gamma_{A}\left[X\right]-\Gamma_{A}\left[X\right]Y\right]=\Pi_{A}\left[X,Y\right]-2\Gamma_{A}\left[\Pi_{A}\left[X,Y\right]\right]A. \end{array}$$

Then, we compute the Lie-bracket

(A14) $$\begin{array}{cc} \left[\tilde{X},\tilde{Y}\right]:=\; & D_{\tilde{X}}\tilde{Y}-D_{\tilde{Y}}\tilde{X}\\ =\; & \tilde{A}\Gamma_{A}\left[dY\left(X\right)-dX\left(Y\right)\right]+\tilde{A}\left(\Gamma_{A}\left[X\right]\Gamma_{A}\left[Y\right]-\Gamma_{A}\left[Y\right]\Gamma_{A}\left[X\right]\right)\\ \; & -\tilde{A}\Gamma_{A}\left[X\Gamma_{A}\left[Y\right]+\Gamma_{A}\left[Y\right]X-Y\Gamma_{A}\left[X\right]-\Gamma_{A}\left[X\right]Y\right]\\ =\; & \tilde{\left[X,Y\right]}+2\tilde{A}\Gamma_{A}\left[\Pi_{A}\left[X,Y\right]\right]A, \end{array}$$

where the last equality in (A14) comes from (A13). Finally, we show the second term in (A14) is vertical. In fact, we have

(A15) $$\begin{array}{c} 2\tilde{A}\Gamma_{A}\left[\Pi_{A}\left[X,Y\right]\right]A=\left(2\tilde{A}\Gamma_{A}\left[\Pi_{A}\left[X,Y\right]\right]{\tilde{A}}^{T}\right)\tilde{A}, \end{array}$$

where $2\tilde{A}\Gamma_{A}\left[\Pi_{A}\left[X,Y\right]\right]{\tilde{A}}^{T}$ is anti-symmetric. Thus, the proof for Lemma A3 has been done. □

By Lemma A3, the proof for Lemma 1 is clarified.

**Proof** **.** (Lemma 1) For any smooth vector field *Z* on SPD(n), and its horizontal lift $\tilde{Z}$, we have

(A16) $$\begin{array}{cc} \; & \langle \tilde{\nabla_{X}Y},\tilde{Z}\rangle =\langle \nabla_{X}Y,Z\rangle \\ =\; & \frac{1}{2}\left(X\langle Y,Z\rangle +Y\langle Z,X\rangle -Z\langle X,Y\rangle +\langle Z,\left[X,Y\right]\rangle +\langle Y,\left[Z,X\right]\rangle -\langle X,\left[Y,Z\right]\rangle \right)\\ =\; & \frac{1}{2}\left(\tilde{X}\langle \tilde{Y},\tilde{Z}\rangle +\tilde{Y}\langle \tilde{Z},\tilde{X}\rangle -\tilde{Z}\langle \tilde{X},\tilde{Y}\rangle +\langle \tilde{Z},\tilde{\left[X,Y\right]}\rangle +\langle \tilde{Y},\tilde{\left[Z,X\right]}\rangle -\langle \tilde{X},\tilde{\left[Y,Z\right]}\rangle \right)\\ =\; & \frac{1}{2}\left(\tilde{X}\langle \tilde{Y},\tilde{Z}\rangle +\tilde{Y}\langle \tilde{Z},\tilde{X}\rangle -\tilde{Z}\langle \tilde{X},\tilde{Y}\rangle +\langle \tilde{Z},\left[\tilde{X},\tilde{Y}\right]\rangle +\langle \tilde{Y},\left[\tilde{Z},\tilde{X}\right]\rangle -\langle \tilde{X},\left[\tilde{Y},\tilde{Z}\right]\rangle \right)\\ =\; & \langle D_{\tilde{X}}\tilde{Y},\tilde{Z}\rangle . \end{array}$$

By the arbitrariness of *Z*, Lemma 1 is proved. □

**Remark** **A2.**
*Proof of Lemma 1 implies that ${d\sigma |}_{\tilde{A}}\left(D_{\tilde{X}}\tilde{Y}\right)$ is independent on $\tilde{A}$ chosen among a fixed fiber.*

## Appendix E. Proof of Theorem 7

**Proof.** Calculating $\tilde{R}{|}_{\tilde{A}}\left(\tilde{X},\tilde{Y},\tilde{X},\tilde{Y}\right)$ as follows

(A17) $$\begin{array}{cc} \; & \tilde{R}{|}_{\tilde{A}}\left(\tilde{X},\tilde{Y},\tilde{X},\tilde{Y}\right)=\langle {\tilde{R}}_{\tilde{X}\tilde{Y}}\tilde{X},\tilde{Y}\rangle \\ =\; & \langle D_{\left[\tilde{X},\tilde{Y}\right]}\tilde{X},\tilde{Y}\rangle -\langle D_{\tilde{X}}D_{\tilde{Y}}\tilde{X},\tilde{Y}\rangle +\langle D_{\tilde{Y}}D_{\tilde{X}}\tilde{X},\tilde{Y}\rangle \\ =\; & \langle D_{\tilde{\left[X,Y\right]}}\tilde{X},\tilde{Y}\rangle +\langle D_{2T(X,Y)}\tilde{X},\tilde{Y}\rangle -\langle D_{\tilde{X}}\tilde{\nabla_{Y}X},\tilde{Y}\rangle \\ \; & -\langle D_{\tilde{X}}T\left(Y,X\right),\tilde{Y}\rangle +\langle D_{\tilde{Y}}\tilde{\nabla_{X}X},\tilde{Y}\rangle +\langle D_{\tilde{Y}}T\left(X,X\right),\tilde{Y}\rangle \\ =\; & \langle \nabla_{\left[X,Y\right]}X,Y\rangle +2\langle D_{T(X,Y)}\tilde{X},\tilde{Y}\rangle -\langle \nabla_{X}\nabla_{Y}X,Y\rangle \\ \; & -\langle T\left(X,Y\right),T\left(X,Y\right)\rangle +\langle \nabla_{Y}\nabla_{X}X,Y\rangle \\ =\; & {R|}_{A}\left(X,Y,X,Y\right)+2\langle D_{T(X,Y)}\tilde{X},\tilde{Y}\rangle -\langle T\left(X,Y\right),T\left(X,Y\right)\rangle , \end{array}$$

where we used (29), (32), $T_{\tilde{A}}\left(X,Y\right)\in V\left(\tilde{A}\right)$, $T_{\tilde{A}}\left(X,Y\right)=-T_{\tilde{A}}\left(Y,X\right)$ and the following equation

(A18) $$\begin{array}{cc} \langle D_{\tilde{X}}T\left(Y,X\right),\tilde{Y}\rangle & =\tilde{X}\langle T\left(Y,X\right),\tilde{Y}\rangle -\langle T\left(Y,X\right),D_{\tilde{X}}\tilde{Y}\rangle \\ \; & =\langle T\left(X,Y\right),T\left(X,Y\right)+\tilde{\nabla_{X}Y}\rangle \\ \; & =\langle T(X,Y),T(X,Y)\rangle . \end{array}$$

Since T(X,Y) and $\tilde{X},\tilde{Y}$ are both vertical, we have

(A19) $$\begin{array}{c} \langle \left[T\left(X,Y\right),\tilde{X}\right],\tilde{Y}\rangle =0. \end{array}$$

Then, using $D_{T(X,Y)}\tilde{X}-D_{\tilde{X}}T\left(X,Y\right)=\left[T\left(X,Y\right),\tilde{X}\right]$ we can obtain that

(A20) $$\begin{array}{c} \langle D_{T(X,Y)}\tilde{X},\tilde{Y}\rangle =\langle D_{\tilde{X}}T\left(X,Y\right),\tilde{Y}\rangle =-\langle T\left(X,Y\right),T\left(X,Y\right)\rangle . \end{array}$$

By the definition of the directional derivative and $\sigma (\tilde{A}+T\left(X,Y\right))=A$, we have

(A21) $$\begin{array}{cc} D_{T(X,Y)}\tilde{X} & =\lim_{t\to 0}\frac{1}{t}\left(\left(\tilde{A}+T\left(X,Y\right)t\right)\Gamma_{A}\left[X\right]-\tilde{A}\Gamma_{A}\left[X\right]\right)\\ \; & =T\left(X,Y\right)\Gamma_{A}\left[X\right]. \end{array}$$

Combining (A20) and (A21), we obtain the following equation

(A22) $$\begin{array}{c} \langle T\left(X,Y\right),T\left(X,Y\right)\rangle =-\langle T\left(X,Y\right)\Gamma_{A}\left[X\right],\tilde{Y}\rangle , \end{array}$$

and

(A23) $$\begin{array}{cc} \tilde{R}\left(\tilde{X},\tilde{Y},\tilde{X},\tilde{Y}\right) & =R\left(X,Y,X,Y\right)+3\langle T\left(X,Y\right)\Gamma_{A}\left[X\right],\tilde{A}\Gamma_{A}\left[Y\right]\rangle \\ \; & =R(X,Y,X,Y)-3\langle T(X,Y),T(X,Y)\rangle . \end{array}$$

Due to $\tilde{R}\equiv 0$, we obtain the explicit expression for the Wasserstein curvature

(A24) $$\begin{array}{cc} R\left(X,Y,X,Y\right)=\; & -3\langle T\left(X,Y\right)\Gamma_{A}\left[X\right],\tilde{A}\Gamma_{A}\left[Y\right]\rangle \\ =\; & 3\mathrm{tr}(\Gamma_{A}\left[X\right]T\left(X,Y\right)\tilde{A}\Gamma_{A}\left[Y\right])\\ =\; & 3\mathrm{tr}(\Gamma_{A}\left[X\right]A\Gamma_{A}\left[\Pi_{A}\left[X,Y\right]\right]A\Gamma_{A}\left[Y\right]). \end{array}$$

□

## Appendix F. Proof of Theorem 8

**Proof.** Using p=r,q≠t, we have

(A25) $$\begin{array}{c} {\left(S_{1}S_{2}\right)}_{ij}=\left(1+\delta^{pq}\right)\left(1+\delta^{pt}\right)\delta_{i}^{q}\delta_{j}^{t}=\left\{\begin{array}{cc} 2\delta_{i}^{q}\delta_{j}^{t}, & q=p\;\mathrm{or}\;t=p\\ \delta_{i}^{q}\delta_{j}^{t}, & q\neq p\;\mathrm{and}\;t\neq p \end{array}\right., \end{array}$$

and

(A26) $$\begin{array}{c} {\left(E_{S_{1}}E_{S_{2}}\right)}_{ij}=\frac{\left(1+\delta^{pq}\right)\left(1+\delta^{pt}\right)}{\left(\lambda_{p}+\lambda_{q}\right)\left(\lambda_{p}+\lambda_{t}\right)}\delta_{i}^{q}\delta_{j}^{t}. \end{array}$$

Then, we obtain

(A27) $$\begin{array}{cc} {\left(\Gamma_{\Lambda }\left[E_{S_{1}},E_{S_{2}}\right]\right)}_{ij} & =\;\frac{{\left[E_{S_{1}},E_{S_{2}}\right]}_{ij}}{\lambda_{i}+\lambda_{j}}\\ & =\;\frac{\left(1+\delta^{pq}\right)\left(1+\delta^{pt}\right)\left(\delta_{i}^{q}\delta_{j}^{t}-\delta_{j}^{q}\delta_{i}^{t}\right)}{\left(\lambda_{p}+\lambda_{q}\right)\left(\lambda_{p}+\lambda_{t}\right)\left(\lambda_{q}+\lambda_{t}\right)}, \end{array}$$

and

(A28) $$\begin{array}{cc} \; & \sum_{k=1}^{n}{\left(\Gamma_{\Lambda }\left[E_{S_{1}},E_{S_{2}}\right]\right)}_{ik}{\left(\Lambda E_{S_{2}}E_{S_{1}}\Lambda \right)}_{kj}\\ =\; & \frac{\left(1+\delta^{pq}\right)\left(1+\delta^{pt}\right)\left(\delta_{i}^{q}\delta_{k}^{t}-\delta_{k}^{q}\delta_{i}^{t}\right)}{\left(\lambda_{p}+\lambda_{q}\right)\left(\lambda_{p}+\lambda_{t}\right)\left(\lambda_{q}+\lambda_{t}\right)}\frac{\left(1+\delta^{pq}\right)\left(1+\delta^{pt}\right)\lambda_{q}\lambda_{t}}{\left(\lambda_{p}+\lambda_{q}\right)\left(\lambda_{p}+\lambda_{t}\right)}\delta_{k}^{t}\delta_{j}^{q}\\ =\; & \frac{{\left(1+\delta^{pq}\right)}^{2}{\left(1+\delta^{pt}\right)}^{2}\lambda_{q}\lambda_{t}}{{\left(\lambda_{p}+\lambda_{q}\right)}^{2}{\left(\lambda_{p}+\lambda_{t}\right)}^{2}\left(\lambda_{q}+\lambda_{t}\right)}\delta_{i}^{q}\delta_{j}^{q}. \end{array}$$

Thus, we obtain the expression for curvature as follows

(A29) $$\begin{array}{cc} {R|}_{\Lambda }\left(S_{1},S_{2},S_{1},S_{2}\right) & =3\mathrm{tr}(E_{S_{1}}\Lambda \Gamma_{\Lambda }\left[E_{S_{1}},E_{S_{2}}\right]\Lambda E_{S_{2}})\\ \; & =\frac{3{\left(1+\delta^{pq}\right)}^{2}{\left(1+\delta^{pt}\right)}^{2}\lambda_{q}\lambda_{t}}{{\left(\lambda_{p}+\lambda_{q}\right)}^{2}{\left(\lambda_{p}+\lambda_{t}\right)}^{2}\left(\lambda_{q}+\lambda_{t}\right)}. \end{array}$$

On the other hand, by (A26), we have

(A30) $$\begin{array}{c} S_{1}\neq S_{2}⟹\langle S_{1},S_{2}\rangle =\mathrm{tr}\left(E_{S_{2}}\Lambda E_{S_{1}}\right)=\mathrm{tr}\left(\Lambda E_{S_{1}}E_{S_{2}}\right)=0, \end{array}$$

and

(A31) $$\begin{array}{cc} \; & \langle S_{1},S_{1}\rangle =\frac{1}{2}\mathrm{tr}\left(S_{1}E_{S_{1}}\right)=\frac{1+\delta^{pq}}{\lambda_{p}+\lambda_{q}},\\ \; & \langle S_{2},S_{2}\rangle =\frac{1}{2}\mathrm{tr}\left(S_{2}E_{S_{2}}\right)=\frac{1+\delta^{pt}}{\lambda_{p}+\lambda_{t}}. \end{array}$$

Then, we can find the area of the sectional parallelogram as

(A32) $$\begin{array}{c} \langle S_{1},S_{1}\rangle \langle S_{2},S_{2}\rangle -{\langle S_{1},S_{2}\rangle }^{2}=\frac{\left(1+\delta^{pq}\right)\left(1+\delta^{pt}\right)}{\left(\lambda_{p}+\lambda_{q}\right)\left(\lambda_{p}+\lambda_{t}\right)}. \end{array}$$

From (A29) and (A32), the proof is done consequently. □

## Appendix G. Proof of Theorem 9

Before proving this theorem, we need to do some preparation. For any A∈SPD(n) with eigenvalues $\lambda_{i},i=1,\cdots ,n$, construct diagonal matrix $\Lambda =\mathrm{diag}(\lambda_{1},\cdots ,\lambda_{n})$ and upper triangle matrix $U={\left(\frac{1}{\lambda_{i}+\lambda_{j}}\right)}_{i<j}$. In p=r,q<t cases, by Theorem 8, we have

(A33) $$\begin{array}{cc} {K|}_{A}\left(S^{p,q},S^{r,t}\right)=\; & \frac{3\left(1+\delta^{pq}\right)\left(1+\delta^{pt}\right)\lambda_{q}\lambda_{t}}{\left(\lambda_{p}+\lambda_{q}\right)\left(\lambda_{p}+\lambda_{t}\right)\left(\lambda_{q}+\lambda_{t}\right)}\\ =\; & 3U_{qt}\left({\left(U+U^{T}\right)}_{pq}\Lambda_{qq}+\delta_{pq}\right)\left({\left(U+U^{T}\right)}_{pt}\Lambda_{tt}+\delta_{pt}\right)\\ =\; & 3U_{qt}\Lambda_{tt}{\left(U+U^{T}\right)}_{tp}\delta_{pq}+3{\left(U+U^{T}\right)}_{pq}\Lambda_{qq}U_{qt}\delta_{pt}\\ \; & +3{\left(U+U^{T}\right)}_{pt}\Lambda_{tt}U_{tq}\Lambda_{qq}{\left(U+U^{T}\right)}_{qp}, \end{array}$$

where we used

(A34) $$\begin{array}{c} \frac{1-\delta^{pq}}{\lambda_{p}+\lambda_{q}}={\left(U+U^{T}\right)}_{pq}, \end{array}$$

and

(A35) $$\begin{array}{c} \frac{\left(1+\delta^{pq}\right)\lambda_{q}}{\lambda_{p}+\lambda_{q}}=\frac{\left(1-\delta^{pq}\right)\lambda_{q}}{\lambda_{p}+\lambda_{q}}\lambda_{q}+\frac{2\delta^{pq}\lambda_{q}}{\lambda_{p}+\lambda_{q}}={\left(U+U^{T}\right)}_{pq}\lambda_{q}+\delta^{pq}. \end{array}$$

Then, we can obtain the scalar curvature by summing (A33).

**Proof.** Note that $U_{qt}=0,q\geq t$, we have

(A36) $$\begin{array}{cc} \rho (\Lambda )=\; & \sum_{p,q<t}{K|}_{\Lambda }\left(S^{p,q},S^{p,t}\right)\\ =\; & \sum_{p,q<t}\left(3U_{qt}\Lambda_{tt}{\left(U+U^{T}\right)}_{tp}\delta_{pq}+3{\left(U+U^{T}\right)}_{pq}\Lambda_{qq}U_{qt}\delta_{pt}\right)\\ \; & +\sum_{p,q<t}\left(3{\left(U+U^{T}\right)}_{pt}\Lambda_{tt}U_{tq}\Lambda_{qq}{\left(U+U^{T}\right)}_{qp}\right)\\ =\; & \sum_{p,q,t}\left(3U_{qt}\Lambda_{tt}{\left(U+U^{T}\right)}_{tp}\delta_{pq}+3{\left(U+U^{T}\right)}_{pq}\Lambda_{qq}U_{qt}\delta_{pt}\right)\\ \; & +\sum_{p,q,t}\left(3{\left(U+U^{T}\right)}_{pt}\Lambda_{tt}U_{tq}\Lambda_{qq}{\left(U+U^{T}\right)}_{qp}\right)\\ =\; & 3\mathrm{tr}(U\Lambda \left(U+U^{T}\right)+\left(U+U^{T}\right)\Lambda U+\left(U+U^{T}\right)\Lambda U\Lambda \left(U+U^{T}\right)). \end{array}$$

□

## Appendix I

**Table A1:** Comparison of three denosing algorithms.

Datasource (Size)	Original SNR	ROR			SOR			AWCD		
		TPR	FPR	SNRG	TPR	FPR	SNRG	TPR	FPR	SNRG
**Stanford Bunny** (10,000)	*10*	***100.00%***	*18.10%*	*452.5%*	*87.78%*	*49.00%*	*79.1%*	***99.28%***	***4.80%***	***1968.3%***
	*0.1*	***100.00%***	*100.00%*	*0.0%*	*84.44%*	***71.61%***	***17.9%***	***99.93%***	*90.53%*	*10.4%*
**Duke Dragon** (100,000)	*100*	***100.00%***	*9.60%*	*941.7%*	*82.29%*	*51.90%*	*58.6%*	***100.00%***	***2.90%***	***3348.3%***
	*1*	***100.00%***	*100.00%*	*0.0%*	*82.12%*	*70.16%*	*17.1%*	***99.88%***	***2.40%***	***4054.8%***
**Armadillo** (50,000)	*10*	***62.26%***	*0.54%*	*114.3*	*88.06%*	*63.46%*	*38.8%*	*34.84%*	***0.01%***	***347.38***
	*1*	***62.66%***	*0.41%*	*153.34*	*87.99%*	*69.62%*	*26.4%*	*34.84%*	***0.09%***	***404.14***
**Lucy** (50,000)	*100*	***100.00%***	*6.00%*	*1566.6%*	*80.71%*	*51.80%*	*55.8%*	***99.99%***	***0.80%***	***123.99***
	*10*	***100.00%***	*35.50%*	*181.7%*	*80.65%*	*66.44%*	*21.4%*	***99.99%***	***2.86%***	***3396.1%***
**Happy Buddha** (50,000)	*100*	***100.00%***	*6.80%*	*1370.6%*	*77.82%*	*56.60%*	*37.5%*	***99.92%***	***2.40%***	***4063.3%***
	*10*	***100.00%***	*13.86%*	*621.5%*	*78.24%*	*64.76%*	*20.8%*	***99.93%***	***1.88%***	***5215.6%***
